# A High-Spin Rate Measurement Method for Projectiles Using a Magnetoresistive Sensor Based on Time-Frequency Domain Analysis

**DOI:** 10.3390/s16060894

**Published:** 2016-06-16

**Authors:** Jianyu Shang, Zhihong Deng, Mengyin Fu, Shunting Wang

**Affiliations:** 1 School of Automation, Beijing Institute of Technology, Beijing 100081, China; 3120130357@bit.edu.cn (J.S.); fumy@bit.edu.cn (M.F.); wstcnt@bit.edu.cn (S.W.); 2 School of Automation, Nanjing University of Science and Technology, Nanjing 210094, China

**Keywords:** spin rate measurement, high-spin projectile, magnetoresistive sensor, time-frequency domain analysis method

## Abstract

Traditional artillery guidance can significantly improve the attack accuracy and overall combat efficiency of projectiles, which makes it more adaptable to the information warfare of the future. Obviously, the accurate measurement of artillery spin rate, which has long been regarded as a daunting task, is the basis of precise guidance and control. Magnetoresistive (MR) sensors can be applied to spin rate measurement, especially in the high-spin and high-g projectile launch environment. In this paper, based on the theory of a MR sensor measuring spin rate, the mathematical relationship model between the frequency of MR sensor output and projectile spin rate was established through a fundamental derivation. By analyzing the characteristics of MR sensor output whose frequency varies with time, this paper proposed the Chirp z-Transform (CZT) time-frequency (TF) domain analysis method based on the rolling window of a Blackman window function (BCZT) which can accurately extract the projectile spin rate. To put it into practice, BCZT was applied to measure the spin rate of 155 mm artillery projectile. After extracting the spin rate, the impact that launch rotational angular velocity and aspect angle have on the extraction accuracy of the spin rate was analyzed. Simulation results show that the BCZT TF domain analysis method can effectively and accurately measure the projectile spin rate, especially in a high-spin and high-g projectile launch environment.

## 1. Introduction

Traditional artillery guidance is an efficient way to enhance the combat efficiency of land-based suppressed weapon systems and minimize the collateral damage [1], as well as the current of development of land weapons in developed countries such as the USA and Russia. China also has done a lot of related research and has made much progress. As is known, accurate measurement of projectile spin rate is the basis of precise guidance and control, a key technology for improving guidance accuracy and flight stability.

Because artillery is firing in a highly dynamic environment, traditional rate sensors are not applicable to the angular rate measurement, especially under the extreme conditions of a high-g (≥12,000 g) and high-spin (≥10 r/s) environment [2]. Compared with the traditional rate sensor, the magnetoresistive (MR) sensor, which can realize high-speed, high-resolution measurement of roll rates, has advantages of passive sensing, small size, high-g survivability, high sensitivity, low power and low cost [3,4,5].

During the ballistic flight, the MR sensor strapped down in the radial direction of the projectile produces a sinusoidally oscillating non-stationary signal. The principle of measuring the projectile spin rate with a MR sensor is based on the corresponding relationship between the frequency of the signal output by the MR sensor and the projectile spin rate. The existing research focused on that theory can be roughly divided into two kinds: some researchers believe that the frequency of the MR sensor output is equal to the projectile spin rate. In [6], Allik *et al.* believed that the frequency of magnetometer output is the same as the spin rate of the mortar. In [7], the projectile spin rate was obtained by extracting the frequency of a SCSA50 magnetic sensor output during each period. Comparing with the Yawsonde spin data, the measurement error is less than 3.6°/s. However, other researchers believe that the frequency of the MR sensor output and the spin rate of projectile are not completely equal. In [8], Harkins *et al.* reported that the magnetic roll rate and projectile spin rate were equal only when there was no yaw motion or the spin axis was perpendicular to the local field vector. In [9], Harkins *et al.* explained that when the spin rate was high with respect to the yaw rate, the projection of the local field vector onto the projectile’s radial axis varies with the spin rate. In [10], Harkins *et al.* also pointed out that when the spin rate was high with respect to the yaw rate and pitch rate, the projectile spin rate estimated by roll period method was more accurate. In this paper, based on the assumption that the spin rate is high with respect to the yaw rate and pitch rate, the relationship between the frequency of the MR sensor output and projectile spin rate was established through a fundamental derivation.

In addition, the frequency of the MR sensor output needs to be extracted to measure the projectile spin rate, so the frequency extraction method of sinusoidally oscillating non-stationary signals is also discussed in this paper. Over the past several decades, researchers have proposed various methods for tracking and extracting signal frequencies. Those can be roughly classified into time domain analysis, frequency domain analysis and TF domain analysis, *etc.*

Each of these methods differs from the others. In the time domain analysis method, the signal period will be estimated to track the signal frequency. The period measurement method used commonly includes peak detection [6,8,9,10,11,12,13] and zero crossing detection [6,10,14,15,16]. In spite of the relatively bigger error, lower sampling rate and the need for sparse data interpolation, time domain analysis method has been widely used in the field of navigation and guidance due to its instantaneity. Frequency domain analysis method, such as Fast Fourier Transform (FFT), Discrete Fourier Transform (DFT) and CZT [17], only has local analytical ability in the frequency domain and is difficult to get the TF information of the time-varying non-stationary signal. With TF domain analysis method, such as Short Time Fourier Transform (STFT) [18], the time and frequency information of time-varying non-stationary signal can be obtained simultaneously. However, the STFT essentially adds the windowed Fourier transform and the precision of the Fourier transform itself is lower, leading to the relatively lower precision of the STFT.

Inspired by STFT, a new BCZT TF domain analysis method is presented in this paper. This method is used to extract the TF information of non-stationary signal whose frequency varies with time. The method is also a very effective validation of projectile spin rate measurement theory and high-accuracy post-processing method. Meanwhile, the BCZT can also be used in a variety of other fields with high-accuracy requirements, such as the rotational speed measurement in industrial and automotive applications. For example, when using MR sensors to measure the rotational speed of the toothed wheel in vehicle anti-lock brake systems, the output of the MR sensor is a sinusoidal signal with varying frequency [19,20,21,22]. Then the high-accuracy angular speed at any time can be extracted by the BCZT. Similarly, the BCZT can also be applied to measure the rotational speed of axes within the gearbox [23]. Furthermore, in the human motion monitoring field, the BCZT can be applied to measure the pedestrian movement frequency, which is a key link in human motion monitoring [24]. When using a MR sensor to estimate the physical activity of a person, the output signal of MR sensor is similar to the sinusoid, and the pedestrian’s accurate movement frequency can be obtained by the BCZT.

The remainder of this paper is organized as means into three main sections plus a conclusion. Section 2 mainly introduces the MR sensor measurement theory of projectile spin rate and its measurement deviation. In Section 3, a signal model whose frequency decays exponentially is constructed. Based on this, the BCZT TF domain analysis method is proposed and its performance is evaluated. In Section 4, an output model of the radial MR sensor is constructed firstly. Then time-spin information is extracted by the BCZT and the effect that launch rotational angular velocity and aspect angle have on extraction accuracy of projectile spin rate is analyzed. Section 5 summarizes the conclusions.

## 2. Mathematical Model

### 2.1. Coordinate Systems and Parameters

**Figure 1:**
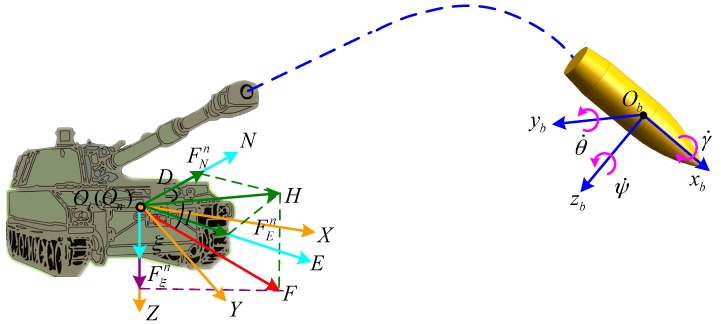
Coordinate systems.

The spin rate measurement system of high-spin projectile includes three coordinate systems (see Figure 1), which respectively are the inertial launch system ($O_{i}XYZ$, *i* system), the navigation-fixed system ($O_{n}NE\xi$, *n* system) and the body-fixed system ($O_{b}x_{b}y_{b}z_{b}$, *b* system).

The inertial launch system is used to describe the 3D coordinates of the projectile centroid relative to the launch site. The origin $O_{i}$ is at the launch site; The *X* axis is parallel to the launch site plane pointing to the target; The *Z* axis is perpendicular to the launch site plane pointing to the ground; The *Y* axis is perpendicular to the *X*-*Z* plane. Navigation-fixed system is chosen to be the north, east, down geographic coordinate system. The origin of the body-fixed system $O_{b}$ is at the center of gravity of the projectile; The $x_{b}$ axis points to the forward direction along the projectile’s longitudinal axis. The $y_{b}$, $z_{b}$ and $x_{b}$ complete the right-handed Cartesian. The $\dot{\gamma }$, $\dot{\psi }$ and $\dot{\theta }$ are spin, yaw, and pitch rates, respectively. 

In addition, *F* is the local geomagnetic field vector of artillery firing spot, *D* is the declination, *I* is the inclination, *H* is the projection of *F* in the horizontal plane, and the projection of *F* into the *n* system is ${\left[\begin{array}{ccc} F_{N}^{n} & F_{E}^{n} & F_{\xi }^{n} \end{array}\right]}^{T}$.

### 2.2. Deriving Projectile Spin Rate with a MR Sensor

The use of MR sensors to measure the projectile spin rate is based on the hypothesis that in most projectile launch ranges, the local field vector *F* is constant [25]. During the ballistic flight, the projection of *F* onto a radially oriented MR sensor’s sensitive axis varies with the projectile spin rate. 

The transformation matrix $C_{n}^{b}$ between navigation frame and body-fixed frame is computed by:

(1) $$C_{n}^{b}=C_{2}^{b}C_{1}^{2}C_{n}^{1}$$

where:

$$C_{n}^{1}=\left[\begin{array}{ccc} \cos\psi & \sin\psi & 0\\ -\sin\psi & \cos\psi & 0\\ 0 & 0 & 1 \end{array}\right],\;C_{1}^{2}=\left[\begin{array}{ccc} \cos\theta & 0 & -\sin\theta \\ 0 & 1 & 0\\ \sin\theta & 0 & \cos\theta \end{array}\right],\;C_{2}^{b}=\left[\begin{array}{ccc} 1 & 0 & 0\\ 0 & \cos\gamma & \sin\gamma \\ 0 & -\sin\gamma & \cos\gamma \end{array}\right]$$

The $\left\{\begin{array}{ccc} \psi & \theta & \gamma \end{array}\right\}$ are yaw, pitch, roll angles, respectively. Using $C_{n}^{b}$ to project *F* into the body-fixed frame:

(2) $$\left[\begin{array}{c} F_{x}^{b}\\ F_{y}^{b}\\ F_{z}^{b} \end{array}\right]=C_{n}^{b}\cdot \left[\begin{array}{c} F_{N}^{n}\\ F_{E}^{n}\\ F_{\xi }^{n} \end{array}\right]$$

where ${\left[\begin{array}{ccc} F_{x}^{b} & F_{y}^{b} & F_{z}^{b} \end{array}\right]}^{T}$ is the projection of *F* into the *b* system at time *t*. Expanding the Equation (2) results in the following three equations:

(3) $$F_{x}^{b}=F_{N}^{n}\cos\psi \cos\theta +F_{E}^{n}\sin\psi \cos\theta -F_{\xi }^{n}\sin\theta$$

(4) $$F_{y}^{b}=B_{1}\sin(\gamma +\beta_{1})$$

(5) $$F_{z}^{b}=B_{2}\sin(\gamma +\beta_{2})$$

where:

(6a) $$B_{1}=\sqrt{{\left(-F_{N}^{n}\sin\psi +F_{E}^{n}\cos\psi \right)}^{2}+{\left(F_{N}^{n}\cos\psi \sin\theta +F_{E}^{n}\sin\psi \sin\theta +F_{\xi }^{n}\cos\theta \right)}^{2}}$$

(6b) $$\beta_{1}=\arctan\frac{-F_{N}^{n}\sin\psi +F_{E}^{n}\cos\psi }{F_{N}^{n}\cos\psi \sin\theta +F_{E}^{n}\sin\psi \sin\theta +F_{\xi }^{n}\cos\theta }$$

(6c) $$B_{2}=\sqrt{{\left(F_{N}^{n}\cos\psi \sin\theta +F_{E}^{n}\sin\psi \sin\theta +F_{\xi }^{n}\cos\theta \right)}^{2}+{\left(F_{N}^{n}\sin\psi -F_{E}^{n}\cos\psi \right)}^{2}}$$

(6d) $$\beta_{2}=\arctan\frac{F_{N}^{n}\cos\psi \sin\theta +F_{E}^{n}\sin\psi \sin\theta +F_{\xi }^{n}\cos\theta }{F_{N}^{n}\sin\psi -F_{E}^{n}\cos\psi }$$

From Equation (3), we can learn that the projection of *F* onto axis $x_{b}$ at time *t*, is correlated with *ψ* and *θ* only, not with *γ*. By Equations (4) and (5) we can see, the projection of *F* onto axis $y_{b}$ and $z_{b}$ at time *t* is similar to sinusoid. Their amplitudes are related to *ψ* and *θ* and phases are related to *ψ*, *θ* and *γ*. The only difference between $F_{y}^{b}$ and $F_{z}^{b}$ lies in different phases. In addition, the rotational angular velocity decays exponentially when traditional artillery flies out bore. According to the modified E. Röggla formula for decaying rotational angular velocity [26], we can write:

(7) $$w(t)=w_{g}e^{\left(-0.4\frac{LD^{3}}{A}t\right)}$$

where, *w*(*t*) is the rotational angular velocity of the projectile at time *t* (rad/s), $w_{g}$ is the rotational angular velocity of the projectile at time *t* = 0 (rad/s), *L* is the width of the projectile (m), *D* is the diameter of the projectile (m) , and *A* is the axial inertia (kg·m^2^). We can then obtain the roll angle γ(t) of the projectile at time *t* by integrating *w*(*t*):

(8) $$\gamma (t)=\int w(t)dt=-\frac{w_{g}A}{0.4LD^{3}}e^{-0.4\frac{LD^{3}}{A}t}$$

Substituting Equation (8) into Equation (4):

(9) $$F_{y}^{b}(t)=B_{1}\sin(-\frac{w_{g}A}{0.4LD^{3}}e^{-0.4\frac{LD^{3}}{A}t}+\beta_{1})$$

where $F_{y}^{b}(t)$ is the sinusoid frequency-modulated wave, modulated by exponential function. The instantaneous phase φ(t) of $F_{y}^{b}(t)$ is solved as:

(10) $$\varphi (t)=-\frac{w_{g}A}{0.4LD^{3}}e^{-0.4\frac{LD^{3}}{A}t}+\beta_{1}$$

Moreover, high-spin projectile is relatively stable during flight, which means the pitch rate and yaw rate are small, so in Equation (6b), *β*_1_ can be roughly approximated as a constant *C*. Therefore, the instantaneous angular rate ω(t) of $F_{y}^{b}(t)$ can now be calculated with:

(11) $$\omega (t)=\frac{d\varphi (t)}{dt}=w_{g}e^{-0.4\frac{LD^{3}}{A}t}$$

The instantaneous frequency (*IF*) f(t) of $F_{y}^{b}(t)$ is then:

(12) $$f(t)=\frac{\omega (t)}{2\pi }=\frac{1}{2\pi }w_{g}e^{-0.4\frac{LD^{3}}{A}t}$$

In addition, utilizing Equation (7), the theoretical spin rate $\dot{\gamma }(t)$ of the projectile at time *t* then is given by:

(13) $$\dot{\gamma }(t)=\frac{w(t)}{2\pi }=\frac{1}{2\pi }w_{g}e^{-0.4\frac{LD^{3}}{A}t}$$

By comparing Equations (12) and (13), we can find the *IF* f(t) of $F_{y}^{b}(t)$ is same as the theoretical spin rate $\dot{\gamma }(t)$. Thus, by extracting the *IF* of $F_{y}^{b}(t)$ at time *t*, we can obtain the projectile spin rate at time *t*.

### 2.3. The Measurement Deviation of the Projectile Spin Rate

From Section 2.2, we learned the *IF* of $F_{y}^{b}(t)$ can reflect the projectile spin rate on the condition that the frequency of $F_{y}^{b}(t)$ is same as the projectile spin rate, that is to say, *β*_1_ is approximated as a constant. However, in practice, *β*_1_ is a function varying with time, which leads to the deviation when solving the projectile spin rate. The actual *IF* of $F_{y}^{b}(t)$ in extracting Equation (4) is $\hat{f}(t)$ by using the TF domain analysis:

(14) $$\hat{f}(t)=\frac{1}{2\pi }\frac{d\varphi (t)}{dt}=\frac{1}{2\pi }w_{g}e^{-0.4\frac{LD^{3}}{A}t}+\frac{1}{2\pi }\frac{d\beta_{1}}{dt}$$

Here, the term $w_{g}e^{-0.4\frac{LD^{3}}{A}t}/2\pi$ in Equation (14) is the theoretical *IF* f(t). By Equation (14), we find that the actual *IF* $\hat{f}(t)$ consists of two parts. If $w_{g}$ is smaller, then $w_{g}e^{-0.4\frac{LD^{3}}{A}t}/2\pi$ is smaller. At this time, $d\beta_{1}/2\pi dt$ cannot be ignored, which will result in bigger deviation in the measurement of the instantaneous spin rate. Only when $w_{g}e^{-0.4\frac{LD^{3}}{A}t}$ is high with respect to $d\beta_{1}/dt$, the impact of $d\beta_{1}/2\pi dt$ on spin rate resolving can be ignored.

In addition, *β*_1_ in Equation (6b) is correlated with the *ψ* and *θ*. If the spin rate measurement system only adopts one MR sensor, then *ψ* and *θ* cannot be determined. Therefore, the measurement deviation of instantaneous spin rate is Δf(t):

(15) $$\Delta f(t)=\hat{f}(t)-f(t)=\frac{1}{2\pi }\frac{d\beta_{1}}{dt}$$

## 3. Tracking Frequency Using BCZT TF Domain Analysis Method

This paper constructs a sinusoidally oscillating frequency modulation signal whose frequency decays exponentially; after analyzing the TF signatures, time domain waveform as well as spectrum signature of the signal, the relation between the manifestation and characteristic parameter of the signal is established, and then the BCZT TF domain analysis method is proposed.

### 3.1. Signal Model

**Figure 2:**
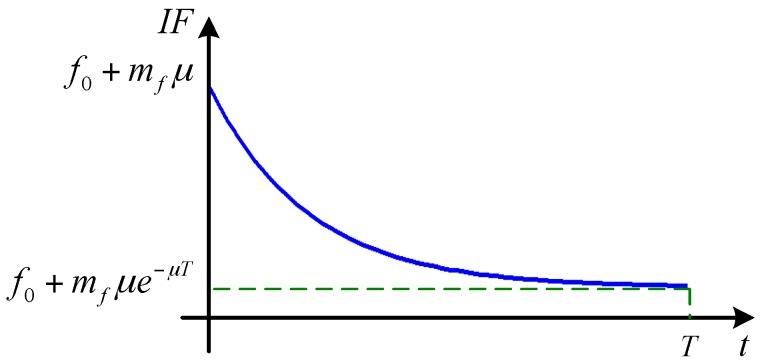
The TF signatures of x(t).

Define the sinusoidally oscillating frequency modulation signal model, whose frequency decays exponentially:

(16) $$x(t)=\frac{A}{2}\sin[\varphi (t)]+x_{0}=\frac{A}{2}\sin[2\pi (f_{0}t-m_{f}e^{-\mu t})]+x_{0}$$

where 0≤t≤T, *T* is the time of signal, *A* is the amplitude, $x_{0}$ is the DC offset, $f_{0}$ is the carrier frequency, *μ* is the modulation frequency, and $m_{f}$ is the modulation index. Since the *IF* is the derivative of the phase of *x*(*t*) , it produces an exponential in the *IF* plane (see Figure 2):

(17) $$IF(t)=\frac{1}{2\pi }\frac{d\varphi (t)}{dt}=f_{0}+m_{f}\mu e^{-\mu t}$$

**Figure 3:**
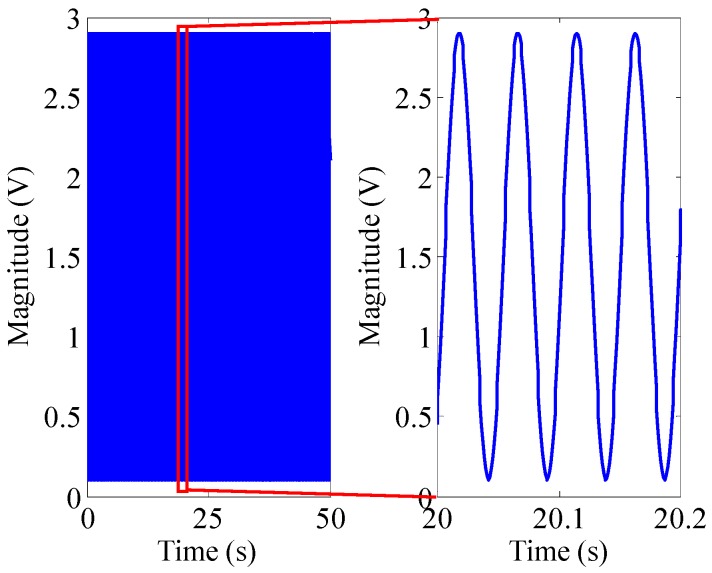
The time domain waveform of *x*(*t*) and its local enlarged drawing.

**Figure 4:**
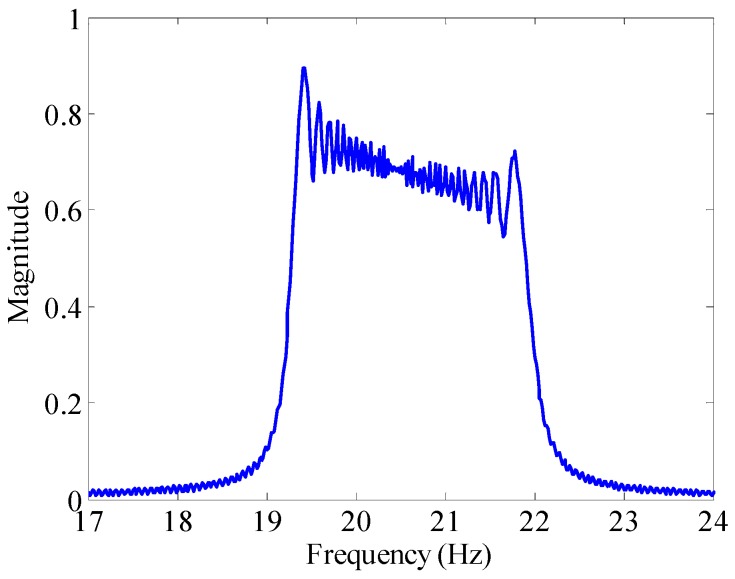
The Fourier spectrum of *x*(*t*).

If we set the time of signal *T* = 50 s, the sampling frequency *f* = 1000 Hz, the amplitude *A* = 2.8 V, the DC offset *x*_0_ = 1.5 V, the carrier frequency *f*_0_ = 15 Hz, the modulation frequency *μ* = 0.01 Hz/s, the modulation index *m_f_* = 700, then the time domain waveform and spectrum of *x*(*t*) are shown in Figure 3 and Figure 4.

From Figure 4, we can find that the amplitude-frequency characteristic of a sinusoidally oscillating frequency modulation signal whose frequency decays exponentially is a range, rather than a determined value, which also indirectly proves that by using a frequency domain analysis method such as the Fourier transform it is difficult to obtain the corresponding frequency information of the signal at any time. The signal with this signature allows TF domain analysis method to obtain time and its corresponding frequency information simultaneously. Therefore, this paper puts forward a BCZT TF domain analysis method to extract the TF information of non-stationary signal.

### 3.2. BCZT TF Domain Analysis Method

**Figure 5:**
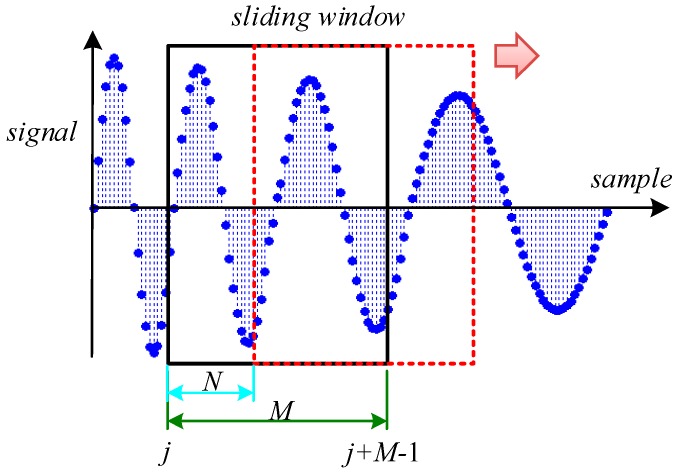
The BCZT TF domain analysis method.

The STFT is a superposed window function on the basis of Fourier transform to track the local feature changes of signals with the assumption that the signal in each window is stationary, yet, the nature of STFT is based on a Fourier transform, and the Fourier transform itself has low resolution, therefore the STFT accuracy is lower. Motivated by the act that STFT is the Fourier transform of the superposed window function, this paper presents a BCZT TF domain analysis method (see Figure 5). 

The BCZT TF domain analysis method adopts CZT with higher frequency resolution than the Fourier transform and uses a Blackman window function with maximum sidelobe leakage to extract accurate TF information of non-stationary signal in Equations (16) and (17).

The non-stationary signal x(t) whose frequency decays exponentially, is modeled by the following equation:

(18) $$x(t)=\frac{A}{2}\sin[2\pi (f_{0}t-m_{f}e^{-\mu t})]+x_{0}$$

The sample x[k] is defined as:

(19) $$x[k]=x(k\Delta t)=\frac{A}{2}\sin[2\pi (f_{0}k\Delta t-m_{f}e^{-\mu k\Delta t})]+x_{0}$$

where *k* is an integer (k≥0), and Δ*t* is the sampling interval. 

Then, we can define a measurement window x[j] containing *M* consecutive samples:

(20) $$X[j]=\left[\begin{array}{cccc} x[k] & x[k+1] & \cdots & x[k+M-1] \end{array}\right]$$

where *j* is the sampling position of the first sample of the measurement window in the entire sequence and j=k+1.

Y[n] is the Blackman window function:

(21) $$Y[n]=\left[\begin{array}{cccc} y(0) & y(1) & \cdots & y(M-1) \end{array}\right]$$

where *M* is the width of Y[n].

y(n) can now be calculated with:

(22) $$y(n)=0.45-0.5\cos\left(\frac{2\pi n}{M}\right)+0.08\cos\left(\frac{4\pi n}{M}\right)\;,\;n=0,1,\ldots ,M-1$$

Multiply *Y*[*n*] by *X*[*j*]:

(23) $$X_{M}[j]=X[j]Y[n]$$

Finally, the frequency of *X_M_*[*j*] extracted by CZT is *f_M_*[*j*], which is also used as the frequency at time (2*j* + *M* − 3)Δ*t*/2 (if *M* is even, time is (2*j* + *M* − 4)Δ*t*/2 ). Moving the *X*[*j*] across the time shaft with velocity *N*, we can get almost all corresponding frequencies of the samples. This is the principle of the BCZT. In addition, some basic properties of the BCZT are as follows:

(1) As *M* is bigger, the frequency resolution is higher, but the time resolution is lower.
(2) (*M*–1) samples’ frequencies cannot be obtained, which results in the deviation of the time domain (*M*–1)Δ*t*. The bigger *M* is, the bigger the deviation is.
(3) *N*, the width between two adjacent measurement windows determines the density of TF information. The larger *N* is, the better real-time performance is.
(4) As is assumed that the corresponding time position *t_M_*[*j*] of the frequency *f_M_*[*j*] is in the middle of the measurement window. Considering the frequency characteristics of the actual signal, time position *t_M_*[*j*] can be adjusted accordingly in order to achieve higher accuracy of TF analysis.
(5) The accuracy of the BCZT is affected by the signal-to-noise ratio (SNR).

### 3.3. Performance Assessment

The analysis of Section 3.2 shows that the accuracy of frequency extracted by the BCZT may be influenced by the width of the measurement window, the corresponding time position *t_M_*[*j*] of the frequency *f_M_*[*j*] in the measurement window, as well as the SNR. These three factors are described in the following sections.

#### 3.3.1. Measurement Window Width

**Figure 6:**
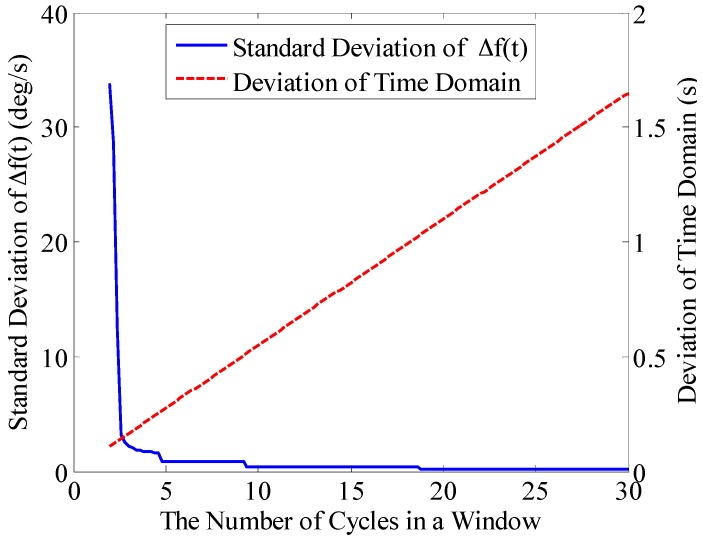
Impact of measurement window width on the accuracy of the BCZT.

Based on the signal model and simulation condition in Section 3.1, the width of the measurement window *M* is set to contain 2–30 periodic signals. The deviation analysis is made between the theoretical frequency *IF*(*t*) and the frequency of *x*(*t*) extracted by the BCZT, and then the standard deviation is obtained and shown in Figure 6. It can be seen that with the increase of measurement window width, the standard deviation of frequency extracted by the BCZT has been decreasing and kept stationary when reducing to a certain extent. However, the deviation of time domain has been increasing all the time. When the measurement window width is less than 2.6 periods, the standard deviation of frequency is bigger (≥10°/s), which results from the leakage of signal energy due to narrow measurement window width. When the measurement window width is more than 3.4 periods, the standard deviation of the frequency is less than 2°/s.

Therefore, in practice, the measurement window width should be selected according to the specific requirements. When the requirement for real-time performance is low, the measurement window width should be increased appropriately to improve the frequency resolution of the BCZT; if the requirement on real-time performance is high, the measurement window width should be narrowed, but not less than three periods.

#### 3.3.2. The Time Position Corresponding to the Frequency in the Measurement Window

From Section 3.2, we can know that frequency *f_M_*[*j*] extracted by the BCZT within the measurement window and its corresponding time position is in the middle of the measurement window. The position of *t_M_*[*j*] can be changed based on the TF signature of the actual data to achieve higher accuracy of TF analysis. The following analysis focuses on the impact of *t_M_*[*j*]’s position within the measurement window on the extraction accuracy of the BCZT.

**Figure 7:**
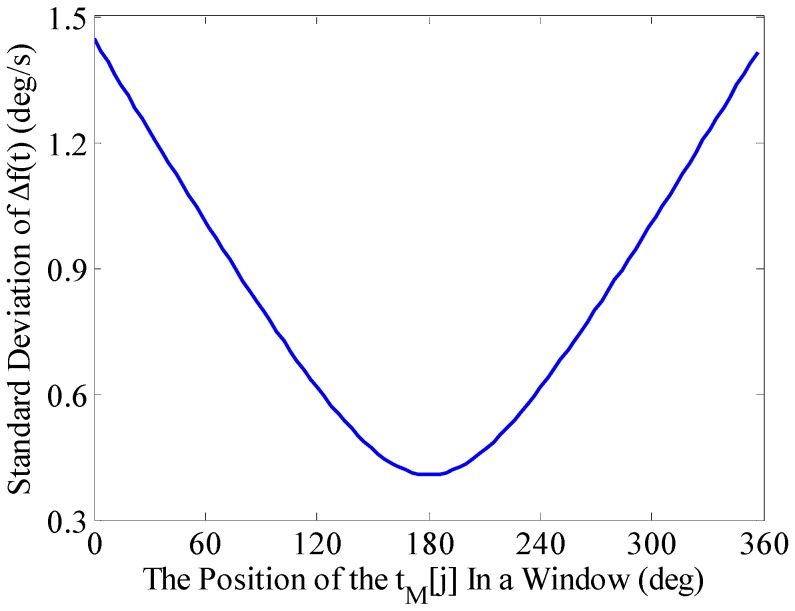
Impact of *t_M_*[*j*]’s position within the measurement window on the accuracy of the BCZT.

Set the measurement window width to contain 18 periodic signals, and then calculate the frequency standard deviation when *t_M_*[*j*] moves from the first point to the last one in the measurement window. The result is shown in Figure 7, where the *x*-axis represents *t_M_*[*j*] × 360°/(*t_j+M–_*_1_ + *t_j_*) × 18 (that is to convert the width of the measurement window to 360°). As can be seen from Figure 7, the frequency standard deviation is smaller when the position of *t_M_*[*j*] is getting closer to the middle of the measurement window and at 178.5° it reaches a minimum 0.41°/s, but not in the middle of the measurement window, *i.e.*, 180°. Therefore, when extracting particular signal frequency, the impact of *t_M_*[*j*]’s position on the accuracy of the BCZT can be pre-analyzed.

#### 3.3.3. SNR

**Figure 8:**
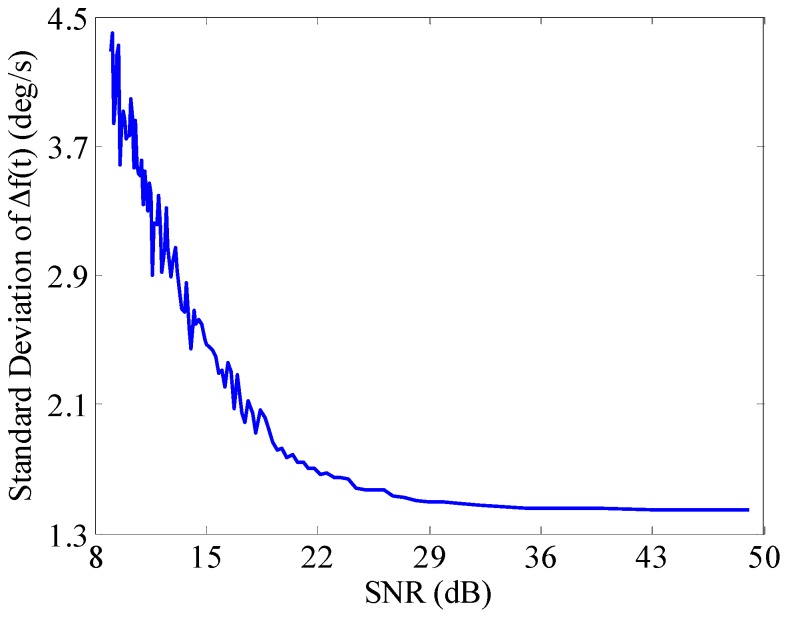
Impact of the SNR on the accuracy of the BCZT.

The changes of the signal amplitude will result in SNR varying in real time. The amplitude of the actual signal changes over time―sometimes it is bigger, sometimes smaller and sometimes it even becomes extremely weak. The SNR becomes larger along with the increased signal amplitude, and *vice versa*. When the amplitude of the useful signal is extremely weak, the SNR is very low and the useful signal may be drowned in the noise signal. Therefore, it is necessary to evaluate the reliability of the BCZT in the aspect of SNR. The following research analyzes the impact of SNR on the accuracy of the BCZT. The SNR ranges from 9–49 dB can be obtained by superimposing noise signal sequentially to the signal *x*(*t*) in Equation (16). The details for setting the SNR ranges between 9 and 49 dB are given in Appendix A. Then, setting the measurement window width to contain 18 periodic signals, using the BCZT to sequentially extract the frequency of the signal and then comparing the extracted signal frequency with the theoretical frequency *IF*(*t*) in Equation (17), the curve of the standard deviation of frequency varying with the SNR is shown in Figure 8.

**Figure 9:**
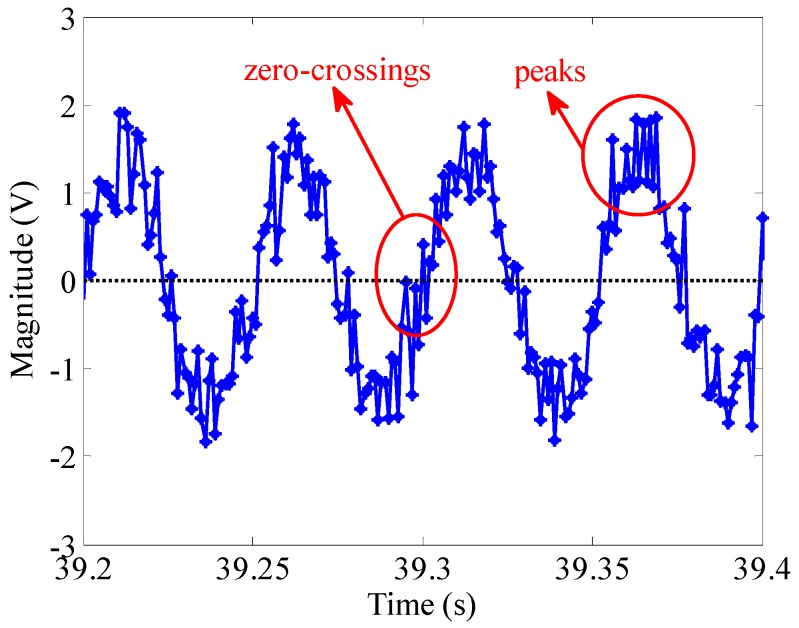
The time domain waveform of *x*(*t*) at a 9-dB SNR.

As can be seen from Figure 8, with the increase of SNR, the standard deviation of frequency is decreasing. When the SNR reaches 9 dB, the noise contained in the signal is bigger and the standard deviation of frequency reaches the maximum 4.4°/s. In this case, due to the noise interference, zero crossing detection and peak detection are almost ineffective (shown in Figure 9), while not only can the BCZT be used, but the deviation is relatively smaller. Therefore, the BCZT has good anti-noise performance, which offers greater advantages in comparison to time domain analysis methods when processing low SNR signals.

## 4. Projectile Spin Rate Estimation

This paper uses the BCZT to extract the frequency of radial MR sensor output to obtain the projectile spin rate. Currently a mathematical model is used to validate the feasibility, which involves the centroid motion equation of traditional projectile, a decaying model of rotational angular velocity of the spinning projectile in ballistic flight (Equation (7)) and a mathematical model of the MR sensor. Although the mathematical simulation is more idealistic than the real experiment, the simulation of the mathematical model can set theoretical values and thus help assess the accuracy and performance of the BCZT.

### 4.1. MR Sensor and Its Mathematical Model

According to the motion characteristics of projectiles and the angle rate measurement requirements, the output characteristics of the three-axis MR sensor HMC1043 are used as a basis for simulation. The size of the HMC1043 is 3 mm × 3 mm × 1.4 mm, the supply voltage is 3 V, the field range is ±6 Gauss, the sensitivity is 1.0 mV/V/Gauss, the resolutions is 120 μGauss and the bandwidth is 5 MHz [27]. Based on the performance parameters of the HMC1043, it can be seen that the sensor’s response frequency can meet the demands of the spin rate measurement for high-spin projectiles. In addition, the HMC1043 is a solid-state sensor that has high-g survivability.

Meanwhile, considering the errors of three-axis MR sensor [28,29] and geomagnetic field, the actual output mathematical model of the three-axis MR sensor can be expressed as:

(24) $$F_{sensor}^{b}=K_{s}K_{n}\left(I_{3\times 3}+M_{i}\right)\left(F^{b}+F_{p}^{b}\right)+F_{0}+F_{n}$$

where, $F_{sensor}^{b}$ is the output of the three-axis MR sensor after various factors being considered; $K_{s}$ is the static sensitivity mismatch error; $K_{n}$ is the non-orthogonality error; $F_{0}$ is the zero-bias; $F_{n}$ is the measurement noise; $I_{3\times 3}$ is a 3 × 3 identity matrix; $M_{i}$ is the inductive magnetic field matrix; $F^{b}$ is the projection of *F* into the *b* system; $F_{p}^{b}$ is the projection of permanent magnetic field vector into the *b* system.

The three-axis MR sensor output model considers the systematic error sources of the three-axis MR sensor and the main magnetic field interference sources (the permanent magnetic field and the inductive magnetic field) when measuring the geomagnetic field. On this basis, the MR sensor output signal for a flying projectile is constructed, which further provides a theoretical basis for the projectile spin rate extraction simulation experiment.

### 4.2. Results and Discussion

**Figure 10:**
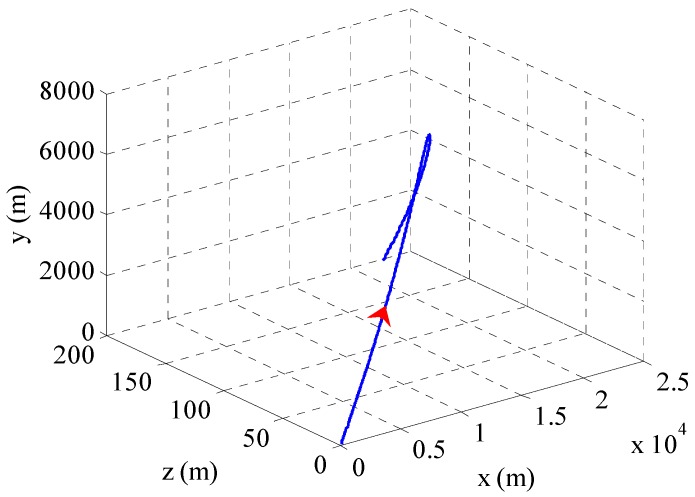
3D trajectory path of projectile in ballistic flight.

**Figure 11:**
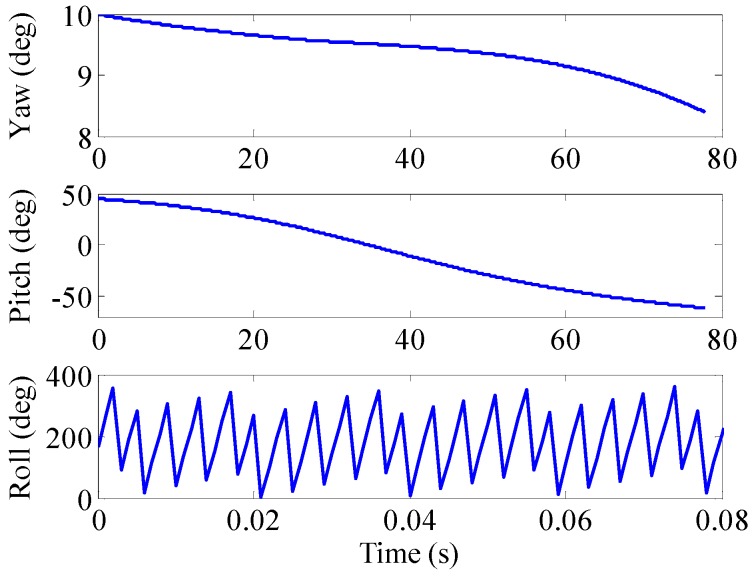
Euler angles of projectile in ballistic flight.

**Figure 12:**
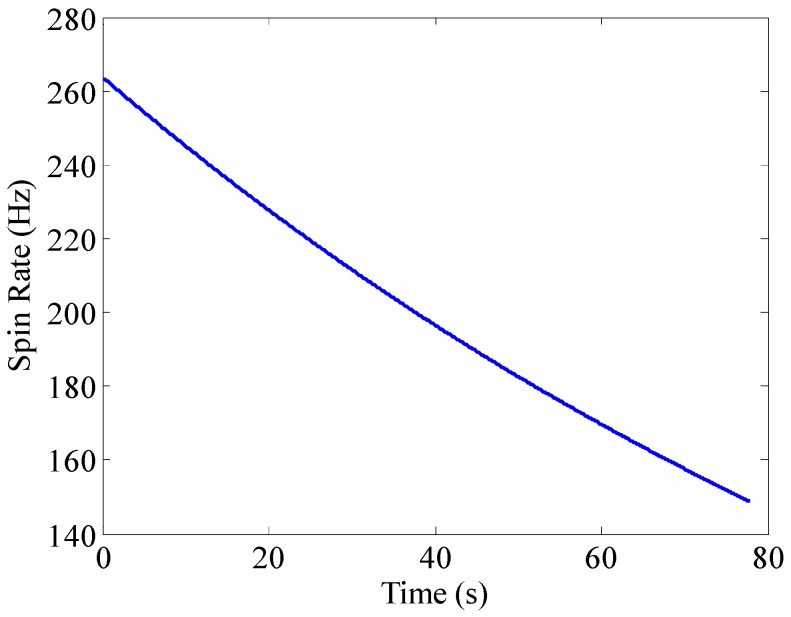
The theoretical spin rate of projectile in ballistic flight.

**Figure 13:**
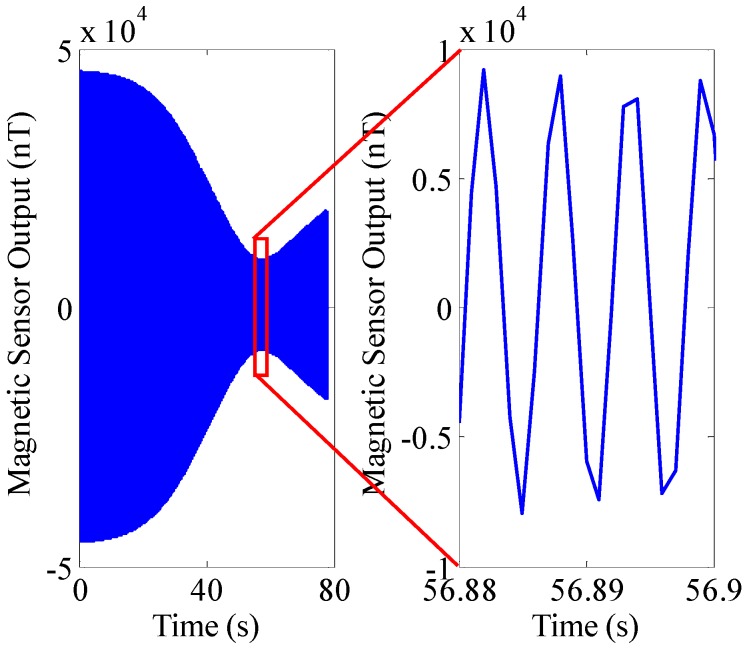
The output of the radially oriented MR sensor from a simulated trajectory. The right figure is a local enlarged drawing of the MR sensor output.

The physical properties of a 155 mm artillery projectile are listed in Table 1. The 3D trajectory path of the projectile in ballistic flight is shown in Figure 10. The ballistic flight reaches 7630.3 m in altitude, flies over 22,493 m downrange and turns to one side 198.04 m. Figure 11 gives the Euler angles of the projectile in ballistic flight. During the flight, the roll angle presents a dense periodic variation. For ease of observation, only part of the roll angle from 0–0.08 s is presented. The theoretical spin rate of the projectile in ballistic flight *f*(*t*) is provided in Figure 12. The *f*(*t*) decays exponentially and slowly from 264–148.78 Hz over the flight. Figure 13 shows the radial MR sensor output and its details, which is similar to a sinusoidal signal with its amplitude decreasing and then increasing.

**Figure 14:**
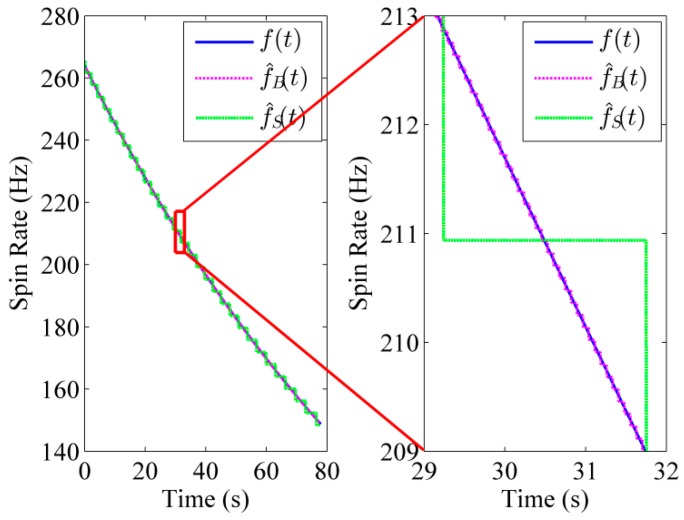
The comparison of theoretical and actual spin rate. The solid blue line, dotted pink line and dashed green line show the theoretical spin rate, actual spin rate extracted by the BCZT and actual spin rate extracted by the STFT, respectively.

When using the BCZT to extract the signal frequency in Figure 13, the measurement window width is set as 1000 samples (deviation of time-domain is 1 s), and the sliding rate of the measurement window is set as a sample point. Figure 14 shows the comparison of extracted TF information ${\hat{f}}_{B}(t)$ of the MR sensor output and the theoretical spin rate *f*(*t*) of the projectile. As can be seen, the actual spin rate ${\hat{f}}_{B}(t)$ is quite close to the theoretical spin rate *f*(*t*) and the standard deviation between them is 12.0°/s (0.013% of the spin rate of the projectile at launch). Meanwhile, STFT [30] is utilized to extract the frequency of the MR sensor output as a comparison method (see Figure 14). The measurement window width of STFT is set as 1000 samples, and the sliding rate of the measurement window is set as a sample point. The standard deviation between the actual spin rate ${\hat{f}}_{S}(t)$ and theoretical spin rate *f*(*t*) is 407.21°/s (0.429% of the spin rate of the projectile in launch). The reason causing bigger error is that STFT essentially adds the windowed Fourier transform and the precision of the Fourier transform itself is lower, leading to the relatively lower precision of the STFT. Thus, the BCZT tracks the spin rate of high-spin projectile with higher accuracy.

### 4.3. The Impact of the Launch Rotational Angular Velocity of the Projectile on Spin Rate Accuracy Extracted by the BCZT

**Figure 15:**
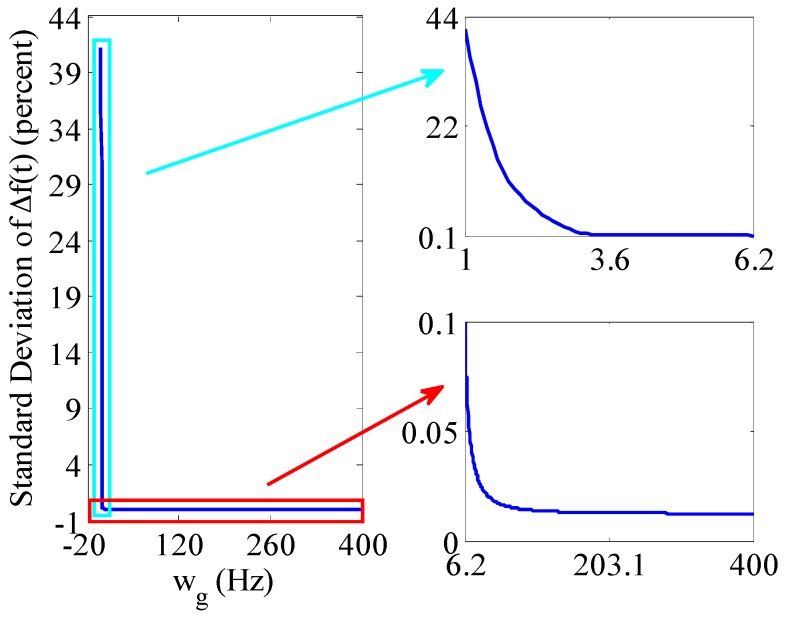
Impact of $w_{g}$ on spin rate accuracy extracted by the BCZT. The top right figure is a local enlarged drawing of spin rate accuracy varying with $w_{g}$ from 1 to 6.2 Hz. The bottom right figure is a local enlarged drawing of spin rate accuracy varying with $w_{g}$ from 6.2 to 400 Hz.

From Section 2.3, we learn that the launch rotational angular velocity of projectile $w_{g}$ has impact on the spin rate accuracy extracted by BCZT. The simulation conditions are set as follows: the measurement window contains 1000 samples, $w_{g}$ varies from 1 to 400 Hz and the results are shown in Figure 15.

The percentage of the standard deviation of the projectile spin rate to $w_{g}$ decreases gradually with the increase of $w_{g}$. When $w_{g}$ ≥ 6.2 Hz, the percentage decreases to less than 0.1%; when $w_{g}$ ≥ 164 Hz, the percentage reduces to less than 0.013%. Therefore, in Section 2, when the measurement principle of spinning projectile based on geomagnetic characteristic is applied to measure the spin rate with a smaller launch rotational angular velocity, the accuracy is lower. However, the bigger the launch rotational angular velocity is, the higher the accuracy is.

### 4.4. The Impact of the Aspect Angle of the Projectile on Spin Rate Accuracy Extracted by the BCZT

It is inevitable to avoid magnetic blind area when using MR sensor to measure the projectile spin rate [31]. When the projectile is launched at a certain aspect angle, the projectile’s *x_b_* axis may be parallel to the local field vector *F* at some time. At this time, the projection of *F* onto the *y_b_* axis is close to zero or extremely tiny, namely a magnetic blind area, which has become a restriction when using geomagnetic information to measure the projectile spin rate.

**Figure 16:**
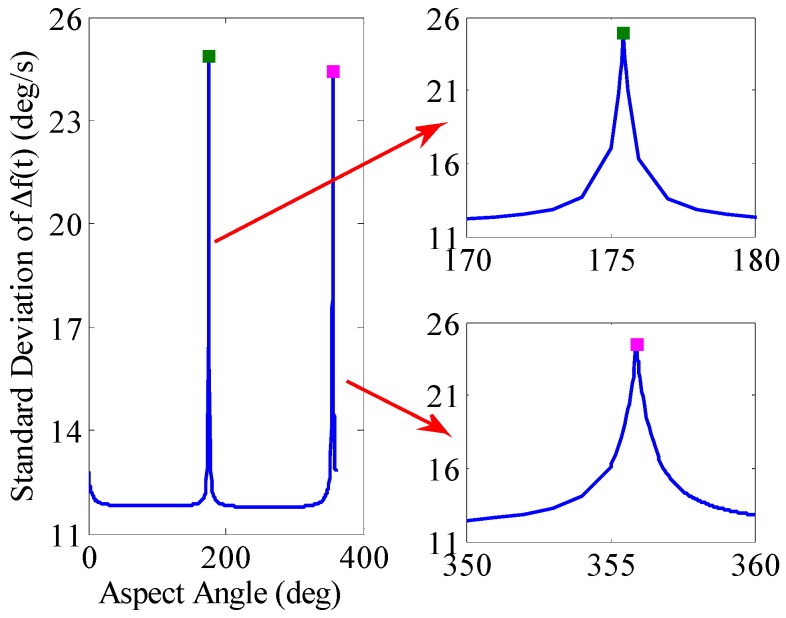
Impact of aspect angle on the spin rate accuracy extracted by the BCZT. The top right figure is a local enlarged drawing of the spin rate accuracy variation with aspect angle from 170° to 180°. The bottom right figure is a local enlarged drawing of the spin rate accuracy variation with aspect angle from 350° to 360°.

To study the impact of aspect angle on the spin rate accuracy extracted by BCZT, the aspect angle is simulated from 0 to 360°. The standard deviation of the spin rate is shown in Figure 16. The result shows that the aspect angle has impact on spin rate accuracy and the standard deviation in most aspect angles is within 12°/s (0.013%); when the aspect angle is 175.45° and 355.91°, the standard deviation of the spin rate reaches to the maximum 24.864°/s and 24.453°/s. It also shows that when the aspect angle is near to *D* + 180° and *D* + 360°, that is, when the *x_b_* axis of the projectile is in the direction of the Earth’s magnetic field or the opposite direction, the standard deviation of the spin rate reaches to the maximum.

**Figure 17:**
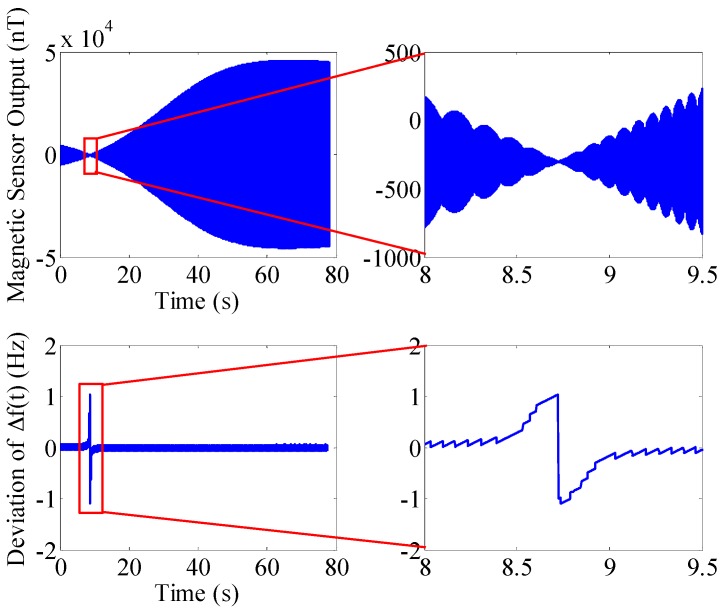
Radial MR sensor output and the spin rate deviation with a launch aspect angle of 175.45°. The top right figure is a local enlarged drawing of the radial MR sensor output. The bottom right figure is a local enlarged drawing of the spin rate deviation with a launch aspect angle of 175.45°.

The following simulation targets the aspect angle in Figure 16, when the standard deviation of the spin rate firstly reaches the maximum, in order to analyze the cause of the above phenomenon. The simulation results are shown in Figure 17. The standard deviation of projectile spin rate during its flight is 24.864°/s with a launch aspect angle of 175.45°. According to Figure 17, although the standard deviation of projectile spin rate during its flight is bigger, only in the period from 8.5–9 s, the deviation of spin rate increases rapidly because during this period, the projectile’s *x_b_* axis is nearly parallel to the local field vector *F*, the projection of *F* on the *y_b_* axis is small. The existing noise interference makes the rotational information of the projectile unreliable, thereby causing the inaccurate spin rate of the projectile, which is a magnetic blind area. Exception for the magnetic blind area, the projection of *F* on the *y_b_* axis is bigger and the projectile spin rate accuracy is higher and more stable. Therefore, when launching the projectile, the aspect angle of the local field vector *F* being perpendicular to the *y_b_* axis should be avoided. In summary, projectile’s spin rate deviation extracted by BCZT is smaller, even when launching the projectile when the standard deviation of the spin rate is bigger. The spin rate deviation only increases rapidly in an extremely short period during its flight, and then decreases rapidly and remains stable.

## 5. Conclusions

The spin rate measurement of projectiles is a key technology in traditional artillery guidance and control. Since the spin rate decays exponentially when the projectile flies out bore, the radial MR sensor produces a sinusoidally oscillating signal whose frequency slowly decays with time. For such a non-stationary signal, this article presents a BCZT TF domain analysis method for the extraction of TF information from a MR sensor output and then gets the projectile spin rate. The simulation results show that the BCZT TF domain analysis method can extract the projectile spin rate accurately. This article draws the following conclusions:

(1) To obtain the establishment conditions of the spin rate measurement principle of the spinning projectile based on geomagnetic information, the launch rotational angular velocity of the projectile should be high enough, and the higher it is, the smaller the deviation of the spin rate measurement is.
(2) The corresponding time position *t_M_*[*j*] of the frequency *f_M_*[*j*] extracted by the BCZT within the measurement window should be determined according to the actual situation. When extracting the signal frequency, the impact that the position of *t_M_*[*j*] in the measurement window has on the accuracy of the BCZT TF domain analysis method can be pre-analyzed in order to determine the best time position.
(3) To define the impact of the measurement window width on the BCZT accuracy, the wider the measurement window is, the higher BCZT accuracy is, but a bigger time domain deviation occurs, so the measurement window width should be selected based on the specific application. When the real-time performance requirement is low, the measurement window width should be appropriately widened to increase the frequency resolution; if real-time performance is highly required, the measurement window width should be as short as possible, but not less than three periods.
(4) Utilizing the BCZT to extract the projectile spin rate is more reliable, even when the projectile is launched in a magnetic blind area. The spin rate deviation increases instantly in a very short period during its flight, then the deviation decreases rapidly and remains stable.

The BCZT TF domain analysis method presented in this paper can also be used to extract the TF information of other non-stationary signals.

## Figures and Tables

**Table 1:** Physical properties of the projectile.

Physical Property	Specification	Physical Property	Specification
Mass, (kg)	46.21	Launch spin rate, (Hz)	264
Width, (m)	0.85	Sampling frequency, (Hz)	1000
Axial inertia, (kg·m^2^)	0.1658	Magnetic field strength, (nT)	44,970
Muzzle velocity, (m/s)	820	Declination, (degree)	−4.85
Quadrant elevation, (degree)	45	Inclination, (degree)	39.12
Aspect angle, (degree)	10	Deviation of time domain, (s)	1

**Figure A1:**
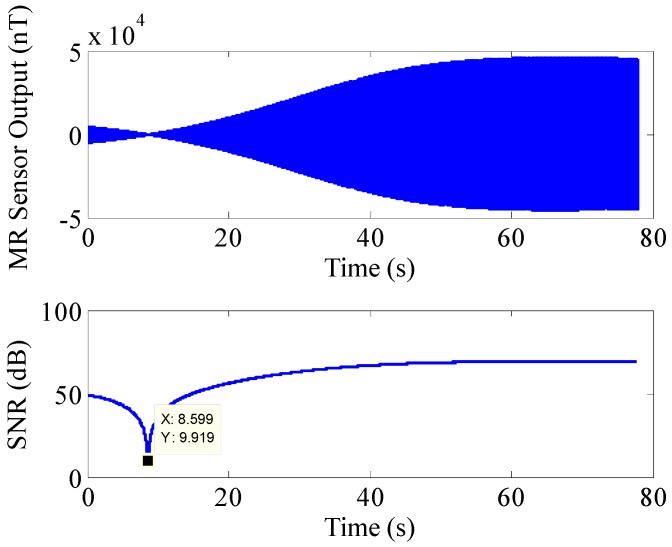
The SNR of the radial MR sensor output with a launch aspect angle of 175.45°.

## Appendix A

In the process of preliminary analysis and signal designing, we set the SNR between 9 and 49 dB based on the noise density of the HMC1043 MR sensor, the possible magnitude of the MR sensor output as well as the research on the possible SNR range emerged in the signal. The calculation for setting the SNR ranges between 9 and 49 dB in Section 3.3.3 is as follows:

(1) Calculate the inherent noise of the HMC1043

The noise power of the HMC1043 can be computed by:

(A1) $$NoisePower={\int_{0}^{Bandwidth}\left(noise\;density\right)}^{2}df$$

where, the noise density of the HMC1043 is 50 nV/$\sqrt{\text{Hz}}$ and the bandwidth is 5 MHz.

Then, the noise voltage of the HMC1043 is solved as:

(A2) $$NoiseVoltage=\sqrt{NoisePower}=1.12\times 10^{-4}V$$

Taking into account the measured magnetic field (MMF) and the supply voltage (SV) of MR sensor, the MR sensor noise corresponding to the calculated noise voltage can be calculated with:

(A3) $$MR_{noise}=NoiseVoltage\times 2\times MMF/SV=1.12\times 10^{-4}V\times 2\times 50000\;nT/3V=3.73\;nT$$

where, $MR_{noise}$ is the MR sensor noise, MMF is distributed on ±50000 nT, and the SV of the HMC1043 is 3 V. Finally, under the condition of 3σ noise standard deviation, the inherent noise of the HMC1043 is 11.19 nT.

(2) Estimate the SNR range of the MR sensor output

As the output of the HMC1043 contains inherent noise, the SNR varies with the useful signal, namely, when the amplitude of the useful signal increases, the SNR increases; when the amplitude of the useful signal decreases, the SNR decreases.

As we all know, a smaller SNR affects the quality of the MR sensor output, which directly influences the spin rate measurement accuracy of the projectile. Hence, an inherent noise of 11.19 nT is applied to calculate the SNR of the MR sensor. On the basis of analyzing the change of the SNR, the range of the SNR can be selected. The SNR ranges are selected for the following reasons: when the projectile is launched with an aspect angle of 175.45°, it flies into the magnetic blind area for a short period of time and the magnetic signal is nearly zero. Meanwhile, the SNR becomes smaller and reaches a minimum value in this area. Figure A1 shows the SNR of the radial MR sensor output with a launch aspect angle of 175.45°; it can be seen that the SNR reaches to the minimum value of 9.9 dB at 8.6 s. Therefore, the range of SNR is set between 9 and 49 dB for analyzing the effect of SNR to the BCZT accuracy, and the SNR range also contains the minimum SNR in the magnetic blind area. In addition, the standard deviation of frequency has no obvious change along with the change of the SNR when the SNR is higher than 49 dB. So the SNR ranges are set between 9 and 49 dB.
